# Rapid autophagic regression of the milk gland during involution is critical for maximizing tsetse viviparous reproductive output

**DOI:** 10.1371/journal.pntd.0006204

**Published:** 2018-01-31

**Authors:** Joshua B. Benoit, Veronika Michalkova, Elise M. Didion, Yanyu Xiao, Aaron A. Baumann, Geoffrey M. Attardo, Serap Aksoy

**Affiliations:** 1 Department of Epidemiology of Microbial Diseases, Yale School of Public Health, New Haven, Connecticut, United States; 2 Department of Biological Sciences, University of Cincinnati, Cincinnati, Ohio, United States; 3 Institute of Zoology, Slovak Academy of Sciences, Bratislava, Slovakia; 4 Department of Biological Sciences, Florida International University, Miami, Florida, United States; 5 Department of Mathematical Sciences, University of Cincinnati, Cincinnati, Ohio, United States; 6 College of Veterinary Medicine, University of Tennessee, Knoxville, Tennessee, United States; 7 Department of Entomology and Nematology, College of Agricultural and Environmental Sciences, University of California Davis, Davis, California, United States; University of Florida, UNITED STATES

## Abstract

Tsetse flies are important vectors of human and animal trypanosomiasis. Ability to reduce tsetse populations is an effective means of disease control. Lactation is an essential component of tsetse’s viviparous reproductive physiology and requires a dramatic increase in the expression and synthesis of milk proteins by the milk gland organ in order to nurture larval growth. In between each gonotrophic cycle, tsetse ceases milk production and milk gland tubules undergo a nearly two-fold reduction in width (involution). In this study, we examined the role autophagy plays during tsetse fly milk gland involution and reproductive output. Autophagy genes show elevated expression in tissues associated with lactation, immediately before or within two hours post-parturition, and decline at 24-48h post-parturition. This expression pattern is inversely correlated with that of the *milk gland proteins* (lactation-specific protein coding genes) and the autophagy inhibitor *fk506-bp1*. Increased expression of *Drosophila inhibitor of apoptosis 1*, *diap1*, was also observed in the milk gland during involution, when it likely prevents apoptosis of milk gland cells. RNAi-mediated knockdown of *autophagy related gene 8a* (*atg8a*) prevented rapid milk gland autophagy during involution, prolonging gestation, and reducing fecundity in the subsequent gonotrophic cycle. The resultant inhibition of autophagy reduced the recovery of stored lipids during the dry (non-lactating) periods by 15–20%. Ecdysone application, similar to levels that occur immediately before birth, induced autophagy, and increased milk gland involution even before abortion. This suggests that the ecdysteroid peak immediately preceding parturition likely triggers milk gland autophagy. Population modeling reveals that a delay in involution would yield a negative population growth rate. This study indicates that milk gland autophagy during involution is critical to restore nutrient reserves and allow efficient transition between pregnancy cycles. Targeting post-birth phases of reproduction could be utilized as a novel mechanism to suppress tsetse populations and reduce trypanosomiasis.

## Author summary

Tsetse flies are vectors for trypanosomes that cause both African sleeping sickness in humans and Nagana in animals. The reduction of tsetse populations is the most efficient way to reduce the prevalence of this economically important disease with current control methods including pesticide application, traps, and sterile insect techniques. Tsetse pregnancy and milk production represent a species-specific target for population control and milk gland transition during each larval growth cycle could represent a novel target for tsetse control. Within one day after birth, the milk gland organ, essential for provisioning nutrients to the intrauterine larva, undergoes involution marked by an ecdysone driven increase in autophagy that allows breakdown of this gland. Inhibiting the process of autophagy prevents the timely transition from the lactation phase to the dry phase, triggering a delay in subsequent pregnancy cycle. This misregulation of milk gland involution leads to an overall decrease in the number of offspring that each female can produce per lifetime. This study has determined the molecular components of this process, and reveals new targets of interference for vector control.

## Introduction

Tsetse are important vectors of disease caused by African trypanosomes, known as Sleeping Sickness in humans and Nagana in animals. An effective way to combat disease involves vector control applications, which are highly efficient due to the low reproductive output of tsetse. Tsetse flies are one of the few insects that employ viviparity [1, 2], characterized by the provision of nutrients beyond egg yolk to support embryonic development [3, 4] and growth of larva to full term within the female uterus. Tsetse females produce a single mature third instar larva during each gonotrophic cycle following a 4–6 day period of intrauterine gestation [1, 2]. Thus, these K-strategists produce only a modest 8–10 progeny per female, per lifetime. [1, 5]. A critical adaptation underlying this reproductive strategy is the modification of the female accessory gland to secrete milk into the uterus for larval consumption. The milk is composed of proteins and lipids emulsified in an aqueous base [6–10]. During lactation, at least 6–10 mg of nutrients dissolved in 12–14 mg of water are transferred to the larva. Molecular characterization of tsetse milk revealed 12 major milk gland proteins, including Transferrin [10], a Lipocalin (Milk Gland Protein1, MGP1 [9,10]), nine tsetse-specific milk proteins (MGP2-3; [8–10]), and Acid Sphingomyelinase 1 (aSMase1; [10]). In addition, peptides involved in mediating immune response, including Ubash3a and Peptidoglycan Recognition Protein-LB (PGRP-LB, [10]), were identified as constituents of tsetse milk. Transcriptomic analysis revealed that the milk proteins represent over 47% of the total transcriptional output in lactating flies. The contribution of the milk protein transcripts declines to less than 2% of the total output two days after the lactation cycle, at parturition [10]. This drastic transcriptional change in milk protein expression indicates a rapid physiological change in the tsetse milk gland following birth.

While previous studies have shown that milk gland involution after birth is quite rapid, with the milk gland shrinking to pre-lactation width in less than one day [11–14], little is known about the mechanisms that underlie this process. Studies on milk gland ultrastructure have shown that lysosome density increases substantially immediately following birth, disappearing completely within two-to-three days [12, 13]. Lysosomes are cellular indicators of milk gland autophagy, but little is known about their regulation and their role in the transition between lactating and non-lactating (dry) periods of the tsetse reproductive cycles. In this study, we examined the role of milk gland involution during the lactating to dry transition, specifically with respect to the autophagic mechanisms employed. We measured transcript abundance for multiple autophagy-related genes and performed knockdown studies on *autophagy related gene 8a* (*atg8a*). Ecdysone application revealed the role of this hormone inducing post-parturition autophagy. Lastly, we used modeling to demonstrate how impaired autophagy could impact the rate of population growth. Our results suggest that autophagy in the milk gland is critical to facilitate the transition between the lactating and dry periods of the tsetse gonotrophic cycle, and that a delay in this autophagic regression reduces fecundity. Given that population reduction is an essential arm of vector control to decrease transmission of parasitic diseases to humans and animals, the ability to alter fecundity through interference with milk gland physiology can expand the tool box for tsetse fly suppression.

## Results

### An increase in autophagic lysosome density precedes milk gland involution

**Fig 1:**
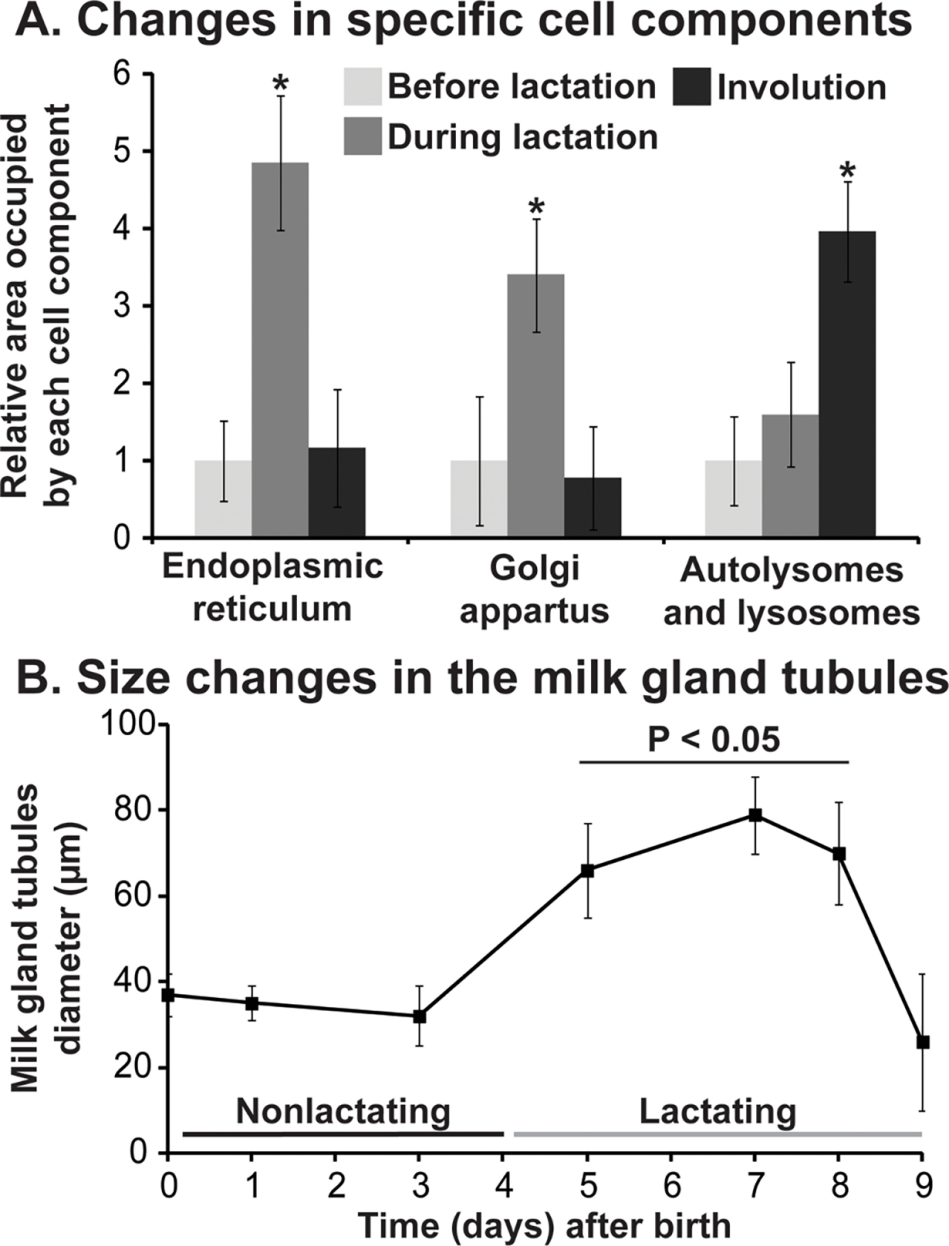
Structural changes in the milk gland during lactation and following involution. A. Relative changes in specific cell components before, during and immediately after lactation. Each bar represents at least 10 separate counts of images from Hecker and Moloo [14] and Denlinger et al. [11]. B. Width of milk gland throughout the pregnancy cycle. Each point represents the width measured from 8 individuals. One-way ANOVA with a post-hoc Tukey test was employed to measure statistical differences. *, indicates significance at P < 0.05.

To assess the level of autophagy at different stages of the gonotrophic cycle, we examined images of the milk gland before lactation, during lactation and immediately after parturition, from previous publications [11–15]. During lactation, a three/five-fold increase in endoplasmic reticulum and Golgi apparatus is noted, while involution is characterized by a nearly 4-fold increase in the number of autolysosomes and lysosomes (Fig 1A). The size of the milk gland reaches its maximum during the peak of milk production, when cells are filled with endoplasmic reticulum, Golgi apparatus, mitochondria, and the milk gland vacuole is fully replete (Fig 1B). The milk gland quickly returns to its pre-lactation state within one day of parturition, following the proliferation of autolysosomes and lysosomes.

### Autophagy genes and apoptosis suppressors are induced during milk gland involution

**Fig 2:**
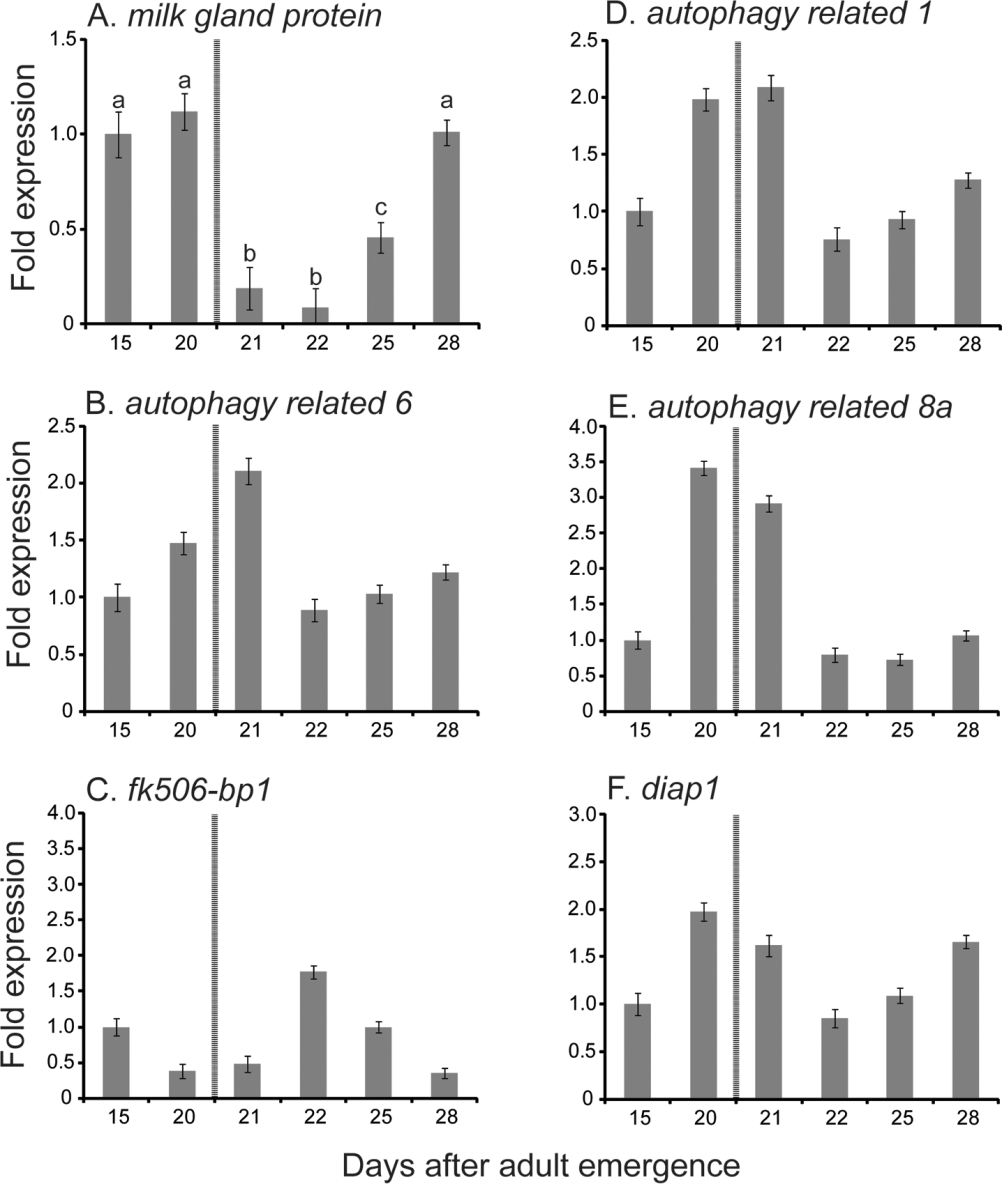
Expression for selected genes involved in lactation and autophagy from *Glossina* in whole flies during late pregnancy and involution. Transcript abundance was determined by qRT-PCR analysis. Line between 20 and 21 days denoted parturition. The data were analyzed with CFX Manager software version 3.1 (Bio-Rad). A-F are specific genes examined. Data represent the mean ± SE for four samples normalized to *tubulin*. a, indicates significantly lower expression than that found in lactating flies. b, indicates significantly higher expression that observed in lactating flies.

**Fig 3:**
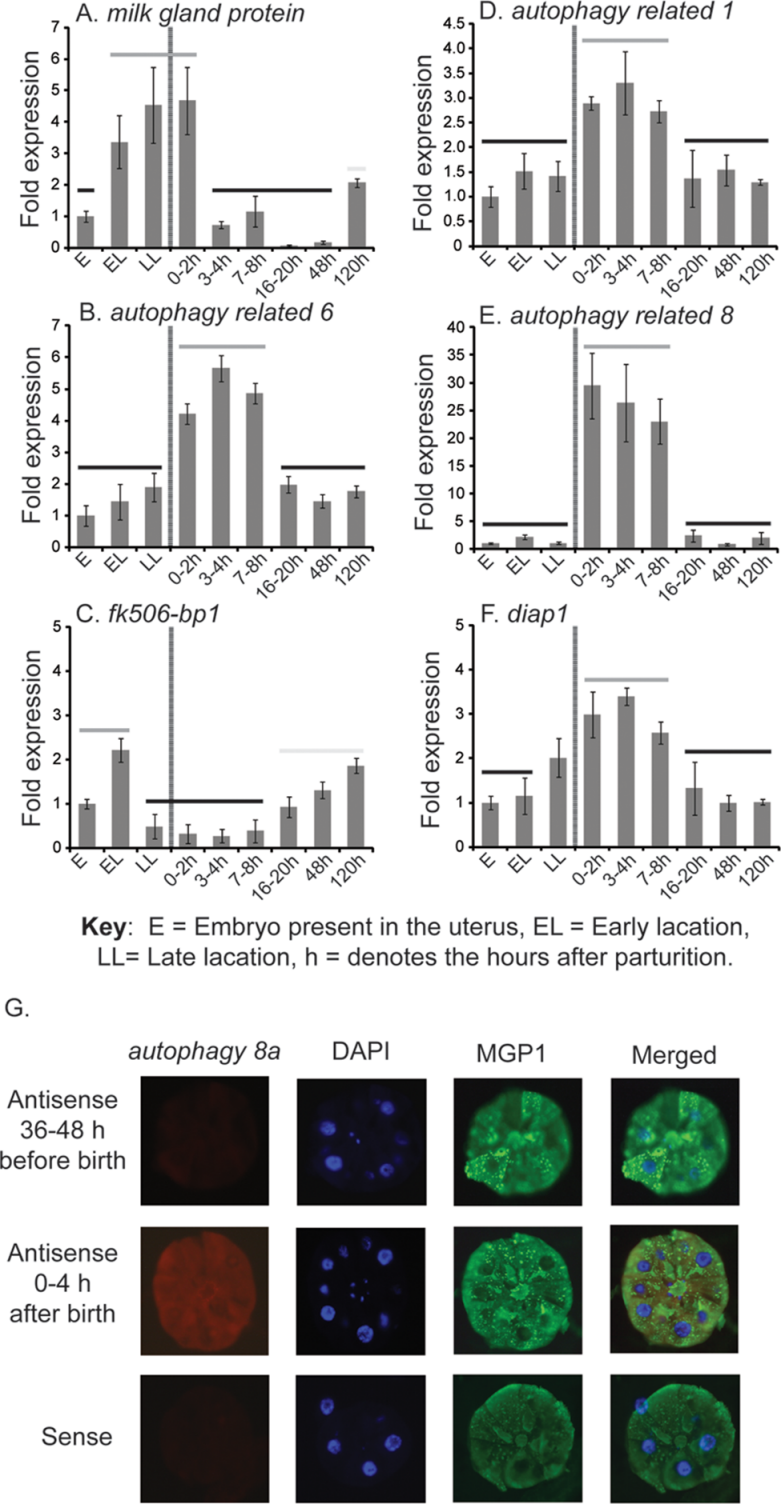
Expression levels of select genes involved in lactation and autophagy from *Glossina* in fat body and milk gland during late pregnancy and involution. Transcript abundance was determined by qRT-PCR analysis. The data were analyzed with CFX Manager software version 3.1 (Bio-Rad). A-F are specific genes examined. Data represent the mean ± SE for four samples normalized to *tubulin*. One-way ANOVA with a post-hoc Tukey test was employed to evaluate statistical differences. Gray lines above the bars indicate significantly higher values than those indicated with a black line. G. *In situ* hybridization of *atg8* 36–48 h before and 0–4 h after birth.

To determine the role of autophagic mechanisms associated with milk gland involution, we measured transcript levels for genes whose products participate in or regulate autophagy [16, 17]. The expression of *autophagy related 1*, *atg1*, (involved in autophagosome induction [17]), *atg6* (involved in autophagosome nucleation) [17], and *atg8a* (involved in autophagosome expansion) [17] was measured during the period encompassing the peak of lactation through 48 hours following parturition (Fig 2). In addition, we monitored expression levels for *fk506-bp1* (an inhibitor of autophagy) [18], and *Drosophila inhibitor of apoptosis 1*, *diap1* (a caspase inhibitor that suppresses apoptosis) [19, 20]. For comparison, we also show transcript levels for a major protein constituent of tsetse fly milk, *milk gland protein 1* (*mgp1*), throughout the course of lactation and involution [10, 21]. In whole female samples, autophagy genes (*atg1*, *atg6* and *atg8a*) show elevated expression during late lactation and parturition, immediately preceding the substantial reduction in *mgp1* expression (Fig 2). *Diap1* has a similar expression profile as *atg* genes, while *fk506-bp1* expression is lowest at end of lactation and 24 hours following larviposition. To more precisely examine the expression of autophagy genes during lactation, we performed fine scale measurements of transcripts in the fat body/milk gland (Fig 3). Complete separation of the milk gland from the fat body into individual tissues is complicated by their intricate physical association [21]. Our results indicate that expression of *mgp1* remains high immediately after parturition (0–2 hours), declines within 3–4 hours following parturition, and then begins to increase again at 120 hours post parturition upon initiation of the next cycle of larvigenesis (Fig 3). *atg1*, *atg6*, *atg8a*, and *diap1* all follow a similar expression pattern in the fat body/milk gland. Expression of these genes remains low during the gonotrophic cycle, except at 0–2 to 7–8 hours after birth (Fig 3). The expression profile of the autophagy inhibitor *fk506-bp1* is inverse of the autophagy genes; lowest expression was observed at 3–4 hours post-parturition (Fig 3). To validate expression in the milk gland, we performed *in situ* hybridization against *atg8a*, confirming *atg8* expression in the milk gland following involution. No differences were noted in *atg8a* expression in the fat body between lactating flies and females immediately after birth (0-2h after parturition). This indicates that the observed differences in expression result from changes in the milk gland and not the fat body.

**Fig 4:**
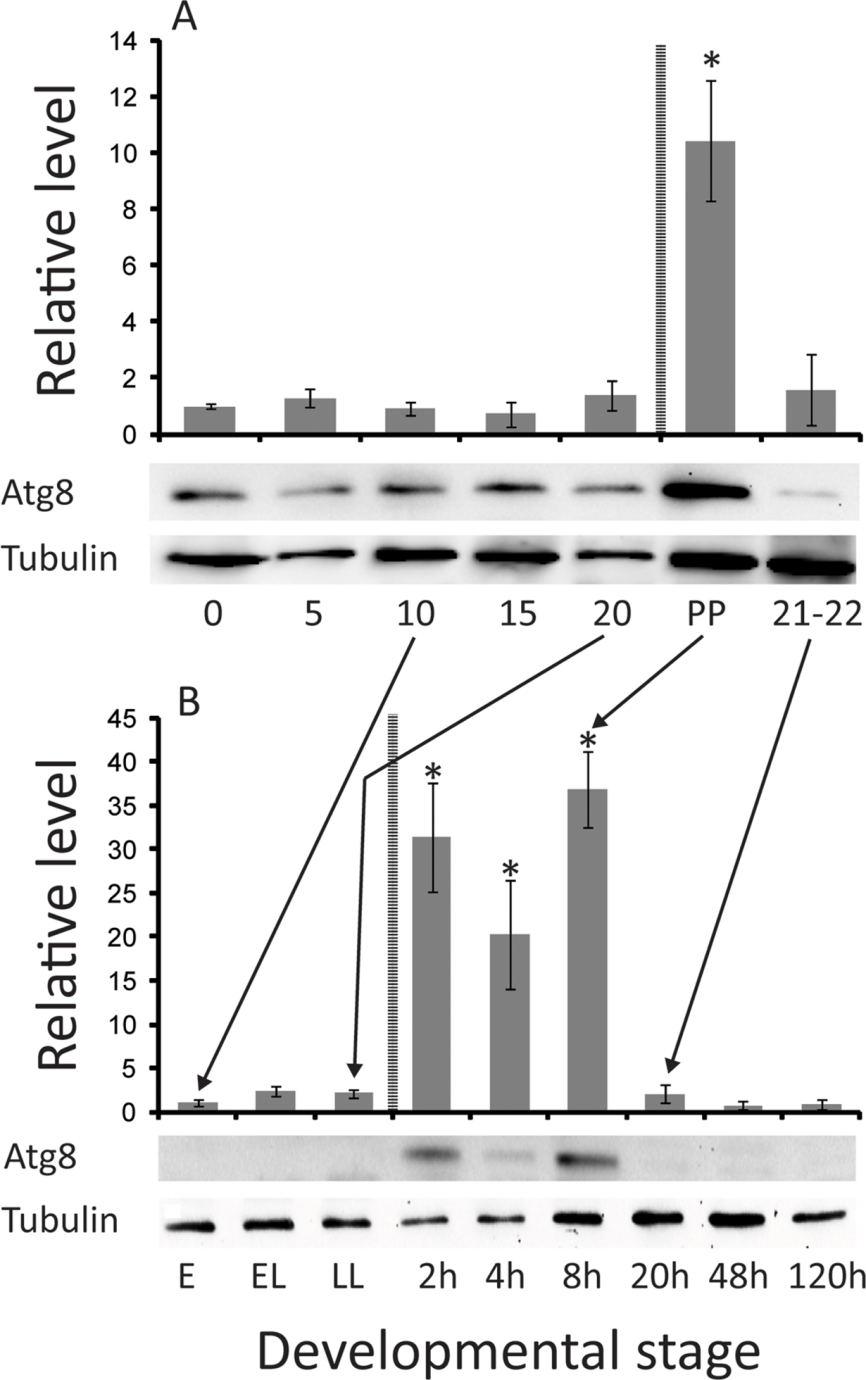
Levels of ATG8 throughout the pregnancy cycle. A. Whole females throughout pregnancy. B. Fat body and milk gland throughout pregnancy. Densitometry was determined based on three replicates using ImageJ. Each bar represents three replicates. One-way ANOVA with a post-hoc Tukey test was employed to evaluate statistical differences. *, indicates significance at P < 0.05.

We additionally examined ATG8 protein levels throughout the tsetse lactation cycle (Fig 4). Analysis of whole body total protein revealed that the ATG8a protein level is increased after birth (post-parturition, Fig 4). When preparations of fat body/milk gland were specifically examined, we observed a sharp increase in ATG8a protein 2-8h after birth, declining to undetectable levels by 20 hours post-parturition (Fig 4). These results correlate with the increase in transcript levels for autophagy-associated genes.

### Suppression of *atg8a* inhibits autophagy and delays subsequent bouts of pregnancy

**Fig 5:**
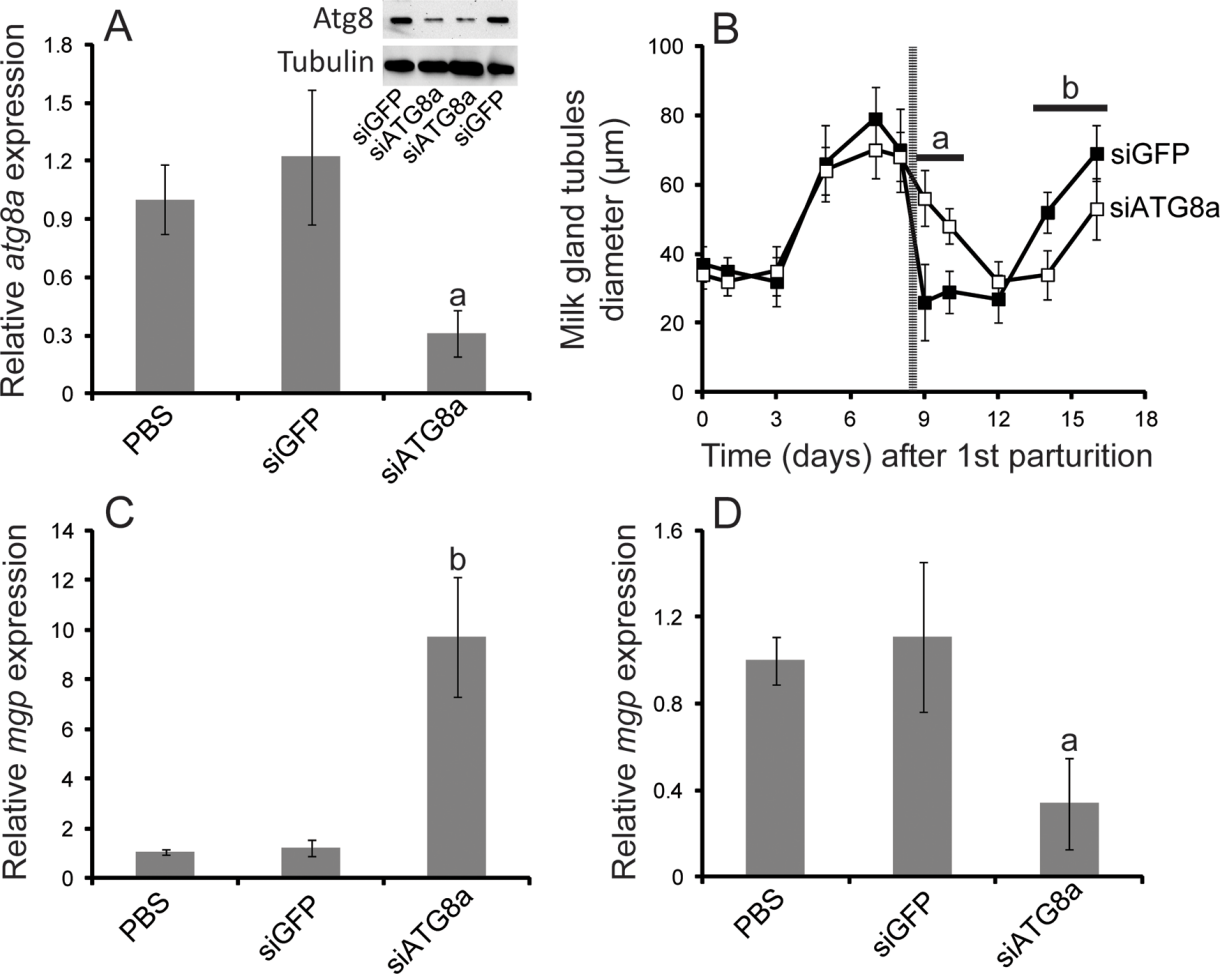
Phenotypic changes following suppression of *atg8a*. A. Transcript and protein (inset) levels following injection of PBS (control), siGFP (control) and siATG8a. Data for qPCR represents the means ± SE for four biological replicates. B. Changes in the milk gland width following injection of siGFP (control) or siATG8a. Data represents the means ± SE for six biological replicates measured in five locations at each time point. C. Expression of *mgp* 24 hours after parturition, as determined by qPCR. Data represents the means ± SE for four biological replicates. D. Expression of *mgp7* days after parturition, as determined by qPCR. Data represents the means ± SE for four biological replicates. One- and two-way ANOVA with a post-hoc Tukey test was employed to evaluate statistical differences. a (low) and b (high), indicates significance at P < 0.05 compared to the PBS-treated control. Line before day 9 denoted parturition.

**Fig 6:**
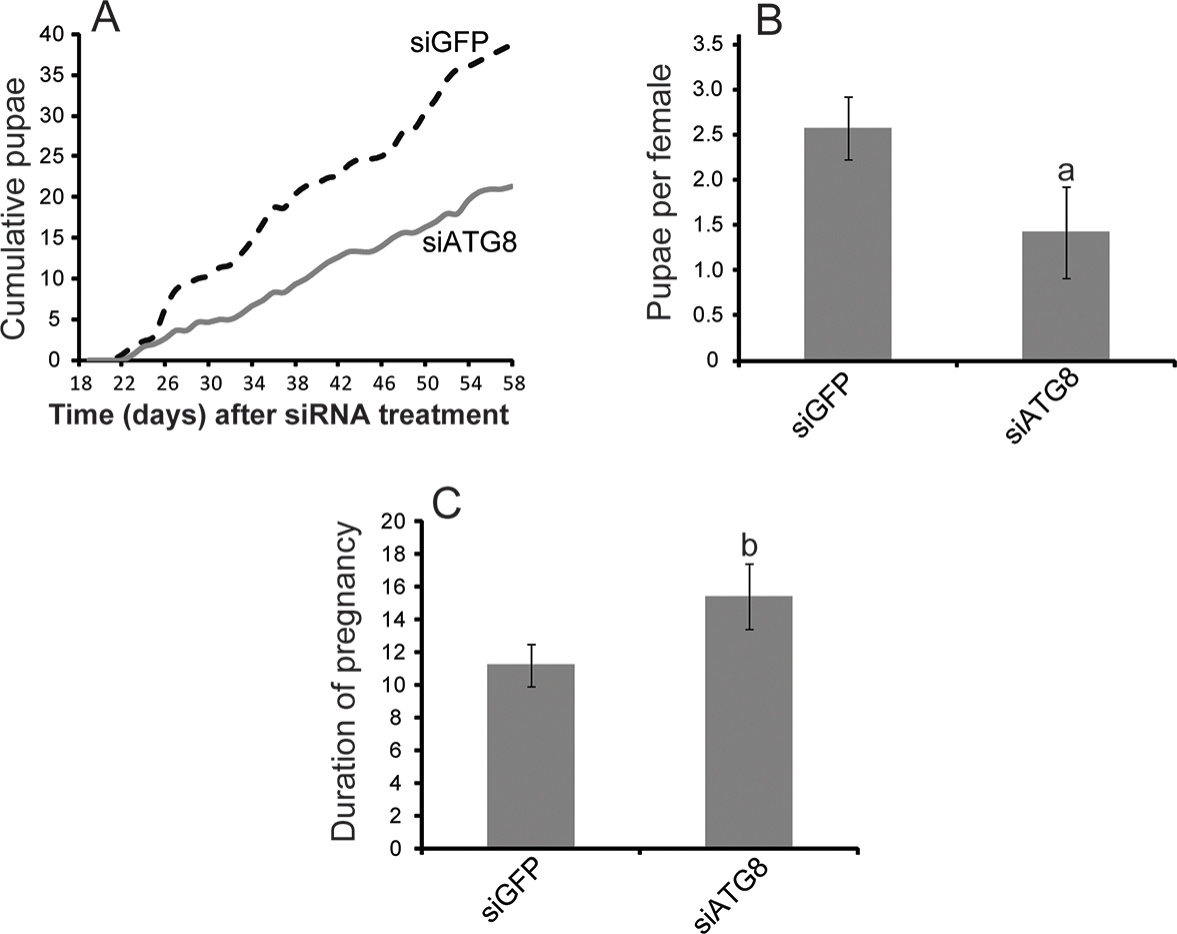
Reproductive output following suppression of *atg8a*. A. Cumulative pupae deposited over the course of 58 days from thirty knockdown or thirty control flies (Two independent replicates) B. Average number of pupae per female and C. duration of pregnancy over 58 days from thirty knockdown or thirty control flies (Two independent replicates). Average duration of pregnancy for each female, over the course of 58 days (1^st^ pregnancy omitted due to longer duration, typically 17–20 days).

To determine the role of ATG8a in milk gland involution, we employed siRNA injection to suppress *atg8a* expression (Fig 5). We were able to reduce *atg8a* expression by nearly 70% compared to control flies, injected with PBS or siGFP (Fig 5). This reduction was also noted at the protein level (Fig 5). Following *atg8a* knockdown, milk gland involution was substantially delayed. Knockdown flies required nearly three days for milk gland morphology to return to the pre-lactation state (as determined by measurement of milk gland tubule diameter) when autophagy was hindered via *atg8a* suppression. In control flies the entire process of involution is usually completed less than 24 hours after birth (Fig 2B). Delayed involution is also evident according to *mgp* expression. *mgp* expression is enriched in siRNA injected flies one day after parturition, a time at when its expression would have otherwise declined to pre-lactation levels (Fig 5). In addition, there is a delay in the usual peak of *mgp* expression during the initiation of the subsequent lactation cycle (Fig 5). We examined fecundity following *atg8a* knockdown and observed a marked decrease in the number of progeny produced (Fig 6A). Thus, fecundity reduction, measured as fewer progeny production per female, is partly a result of an extended pregnancy cycle due to the lack of optimal nutrients to support larval growth (Fig 6B and 6C).

### Ecdysone injection promotes milk gland autophagy in pregnant females

**Fig 7:**
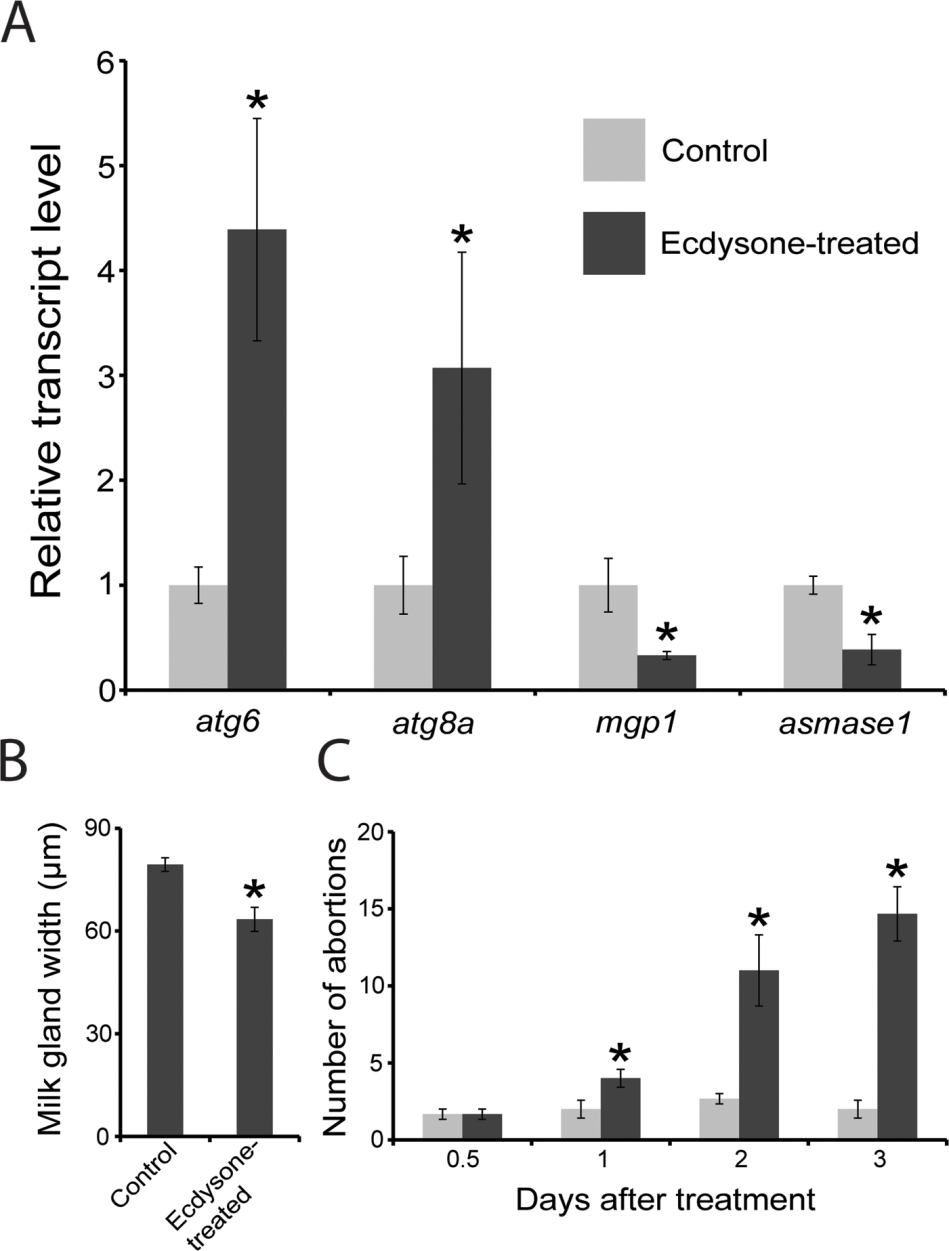
Effect of ecdysone injection on milk gland autophagy. A. Expression of two milk protein genes (*milk gland protein*, *mgp*, and *acid sphingomyelinase 1*, *asmase1*) and two autophagy genes (*atg6* and *atg8a*); each bar represents three replicates of two combined individuals B. Width of milk gland, each bar represents 12 individuals). C. Number of abortions observed following ecdysone treatment; Each bar represents 3 biological replicates of 50 individuals. One-way ANOVA with a post-hoc Tukey test was employed to evaluate statistical differences. *, indicates significance at P < 0.05.

Previous studies have documented a substantial increase in ecdysone titer immediately preceding parturition [22], which declines to baseline levels within 24 hours. To determine if initiation of milk gland autophagy is associated with the high levels of ecdysone observed around parturition, we applied ecdysone at physiologically-relevant levels to whole females and observed a significant increase in both autophagy associated gene expression (*atg8a* and *atg6*) and a reduction in *mgp* and *asmase1* expression (Fig 7A). This milk protein gene suppression correlates with a decrease in milk gland diameter following ecdysone treatment (Fig 7B). Furthermore, ecdysone treatment increased the rate of larval abortion within 2d post treatment, and even more dramatically 3-4d after treatment (Fig 7C). This delay likely results from impaired milk gland function, leading to larval malnutrition or starvation. These data suggest that the ecdysone peak immediately preceding larviposition likely contributes to the induction of milk gland autophagy that follows parturition.

### Population growth rate after interference with autophagy

**Fig 8:**
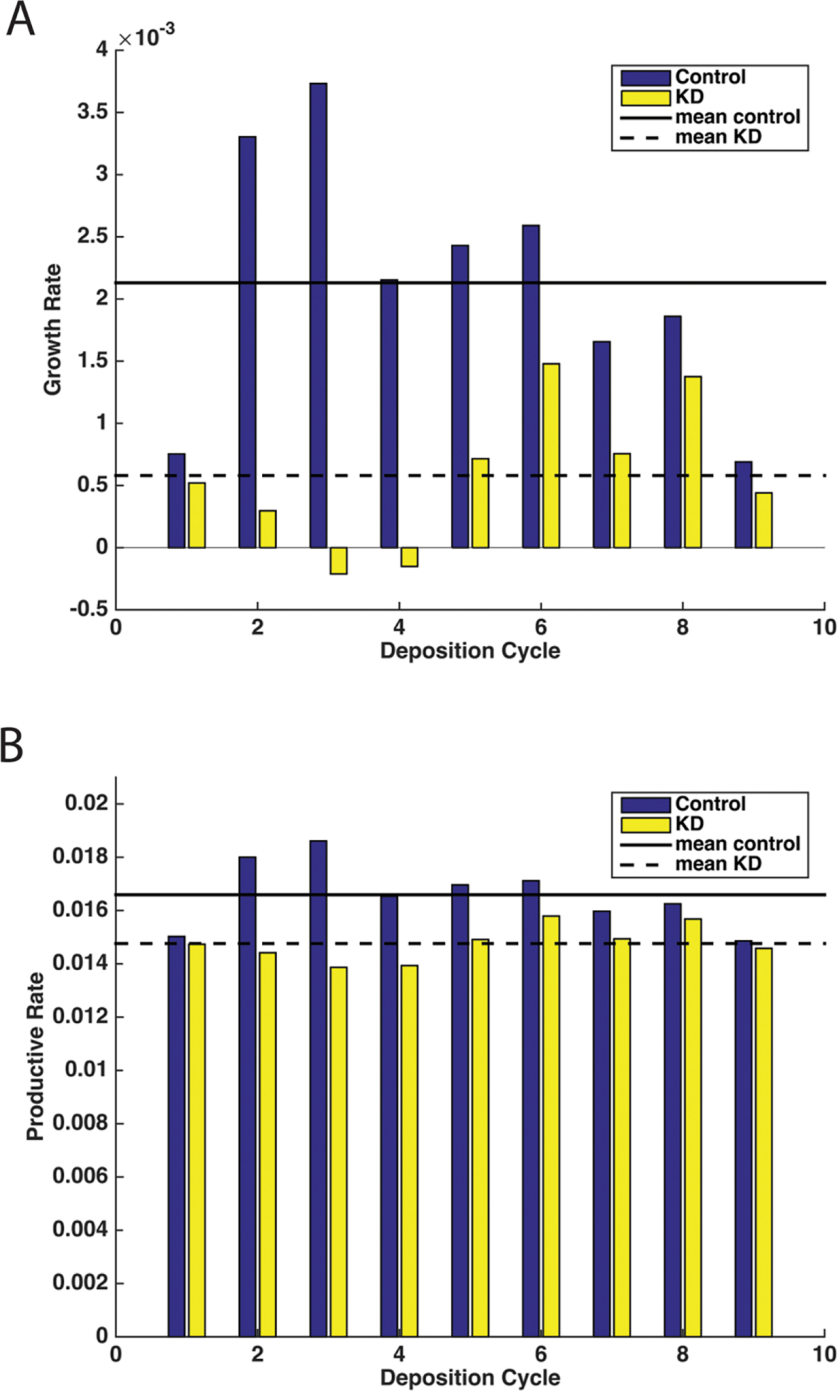
Population modeling following *atg8a* knockdown. A. Frequency of growth rate. B. Reduction in productive (= growth) rate between *atg8a* knockdown (KD, siATG8) and control (siGFP). Results represent 10,000 simulated replicates.

To assess the effect of involution associated autophagy disruption on tsetse population dynamics, we modeled the potential effects this would have on population growth following impaired autophagy following the first birth. Based on the modeling analysis, reproductive growth rate was reduced by 75% during the entire lifetime of a female when the process of milk gland involution was impaired following *atg8a* knockdown (Fig 8A). The greatest reduction in reproductive growth rate occurred in early gonotrophic cycles (cycles 2–5), when female reproductive output is the highest (Fig 8A). In addition, the population growth rates were reduced by ~15% following *atg8a* knockdown when compared to those injected with siGFP (Fig 8B). Similar to the reduction in reproductive growth rate, the most substantial difference in the population growth rates occur during the early gonotrophic cycles. Overall, these modeling results indicate that impaired milk gland autophagy will reduce population maintenance of tsetse flies.

## Discussion

**Fig 9:**
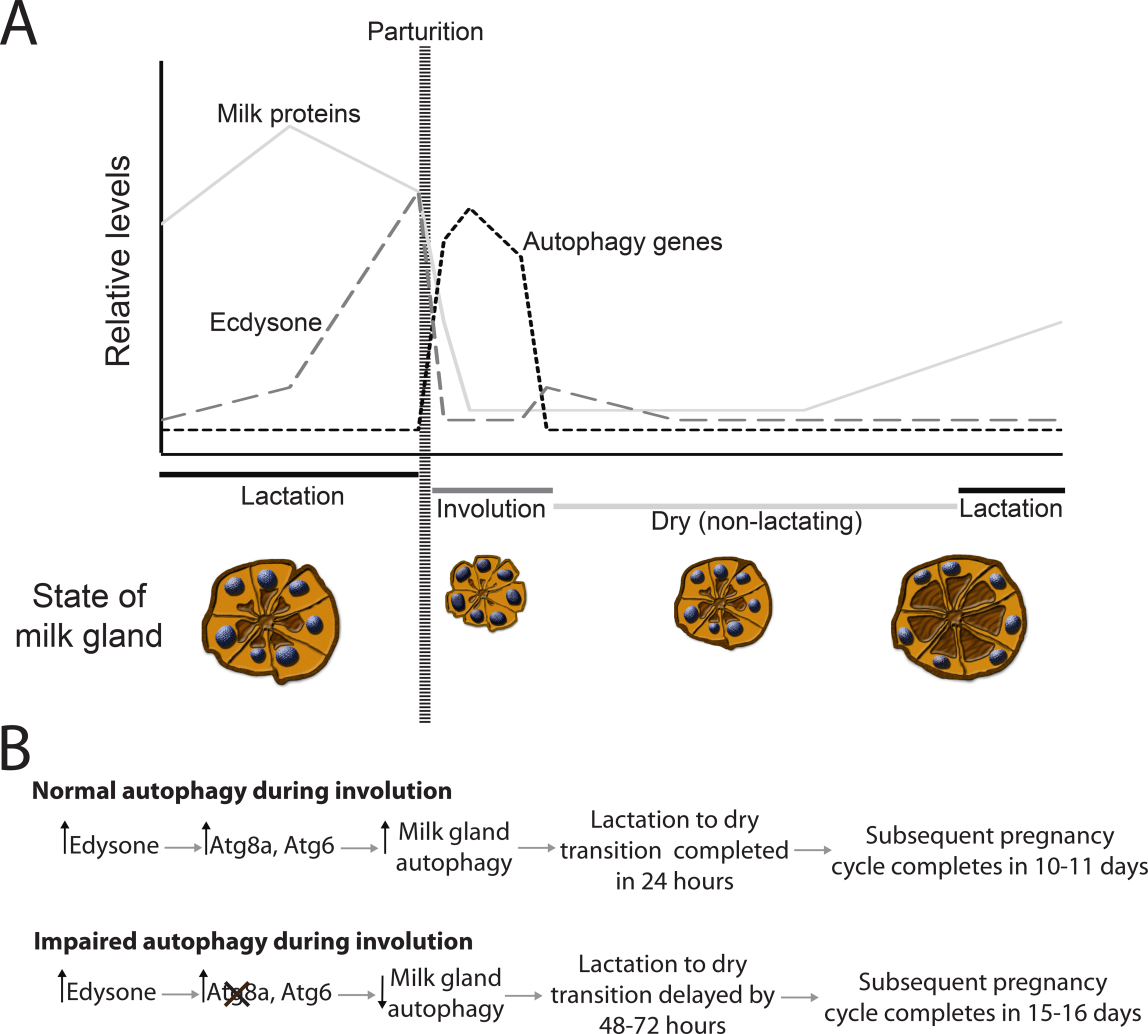
Summary for autophagy associated with tsetse fly involution. Cross sections adapted (A) in relation to changes of autophagy-associated proteins (B) based on Ma and Denlinger [15], Hecker and Moloo [14], Yang et al. [9], and Michalkova et al. [43].

In this study we show that milk gland remodeling between cycles of lactation is critical to maintain optimum reproductive capacity in tsetse. Milk gland involution usually completes within one day after parturition. Expression patterns for autophagy genes suggest that milk gland remodeling likely relies on autophagy rather than apoptosis or necrosis. Suppression of autophagy and consequently involution delayed the subsequent pregnancy cycle by 2–3 days rather than 24 hours. An ecdysone peak that occurs immediately preceding parturition likely triggers autophagic processes in the milk gland. Hindering autophagy reduces the reproductive rate, which could lead to a decrease in population growth rate below replacement levels, indicating that this is a crucial aspect of the tsetse reproductive cycle. We have provided a summary illustrating the role of autophagy during involution during tsetse fly pregnancy (Fig 9).

Autophagic mechanisms during insect reproduction have been examined in the mosquito, *Aedes aegypti* [23]. In many mosquito species, oogenesis is directly dependent on blood feeding [24, 25]. Acquisition of a bloodmeal triggers vitellogenesis, during which the fat body produces a massive amount of yolk protein precursors that subsequently accumulate in the developing oocytes [24, 25]. This process is regulated by ovarian ecdysiotropic hormone (OEH), an insulin-like peptide produced in a subset of neurosecretory cells, which triggers the production of 20-hydroxyecdysone (20E) [26–28]. An increase in the 20E titer, along with nutritional stimulation by amino acids through the target of rapamycin (TOR) signaling pathway, is responsible for the initiation and maintenance of vitellogenesis in mated, blood fed mosquitoes [29, 30]. After oviposition, yolk protein gene expression is drastically reduced coinciding with an increase in lysosomal activity in the fat body [23]. Recently it was demonstrated that this rapid decline in expression of yolk proteins involves programmed autophagy that is regulated through ecdysone signaling [23]. In particular, a reduction in autophagy that occurs after vitellogenesis leads to a prolonged expression of Vg, a yolk protein gene, beyond what is necessary for egg production [23] leading to retardation of egg development in subsequent reproductive cycles [23]. This process is similar to that observed in tsetse, in which we have shown that autophagic regression of the milk gland is critical for subsequent reproductive cycles; impaired autophagy delays subsequent pregnancy cycles. While the effect reported here is moderate compared to the observations in *A*. *aegypti* [23], it is clear that programmed autophagy, following the completion of the reproductive cycle, is a critical and conserved mechanism to ensure optimum fecundity for insects.

Previous studies in tsetse revealed a rapid increase in circulating ecdysteroid immediately before parturition [22, 31], and application of ecdysteroid induces larval abortion in pregnant mothers [31, 32]. Interestingly, it was an increase in ecdysone, rather than 20-hydroxyecdysone, that immediately preceded parturition [22, 31]. This distinction is notable; in the uterus contractions are induced by ecdysone but not by 20-hydroxyecdysone [33]. Therefore, in this study we tested the effects of ecdysone on milk gland autophagy. Our results indicate that ecdysone induces the expression of autophagy genes in both whole body and decreases milk gland width. In mosquitoes, autophagy in the fat body at the termination of vitellogenesis requires ecdysteroid signaling, as suppression of the ecdysone receptor (EcR) was demonstrated to inhibit this process [23]. The observation of ecdysone mediated cyclical autophagic responses to changing reproductive states in both tsetse and mosquitoes suggests that this may be a conserved mechanism in Diptera.

Recent studies have suggested that tsetse lactation is analogous to lactation in mammals as evidenced by a number of similarities between the two systems [10, 34]. These similarities include clearly defined dry (non-lactating) and lactating periods [1], transfer of beneficial bacteria via milk secretions [35, 36], aquaporin mediated water transfer for milk hydration within lactation associated tissues [7] and presence of functionally analogous proteins in tsetse and mammalian milk. These milk proteins include iron-binding proteins (lactoferrin in mammals and transferrin in tsetse [37, 38]), lipocalins and an expanded gene family coding for caseins in mammals and MGP2-10 in tsetse [10, 39, 40] that serve as both a protein resource and a critical source of phosphates.

Following involution in mammals, tissue breakdown occurs via a combination of autophagic, apoptotic and necrotic mechanisms. The tissue is then repopulated with adipocytes until the next period of lactation [41, 42]. Thus, the transition between dry and lactating periods in mammals involves a drastic shift in the type and amount of specific cells. For tsetse, milk gland involution likely does not involve apoptosis or necrosis. Rather, the process is limited to autophagic regression, likely because milk gland regeneration must begin within one day [1, 12, 14], where a complete cellular breakdown and regrowth would require longer periods between bouts of lactation. This slower recovery after birth would lead to extended pregnancy, which in tsetse will reduce lifetime female fecundity, causing the population growth rate to drop below replacement levels [43, 44]. Therefore, although tsetse and mammals share many analogous lactation-related phenomena, involution mechanisms differ due to the rapid rate at which tsetse must initiate subsequent lactation cycles to ensure fecundity.

In conclusion, we provide the first analysis of the molecular mechanisms underlying milk gland involution. We show that milk gland involution is completed within the 24 hours following parturition and is subject to autophagic mechanisms. This likely occurs without cellular apoptosis or necrosis, allowing the cells to increase in size during milk production and undergo autophagic regression during involution. Interference with milk gland autophagy prevents a timely switch from lactating to nonlactating state, leading to lower reproductive output in subsequent generations, and a predicted failure to maintain population growth above what is necessary for population replacement. This project complements previous studies that showed that both dry [10, 45] and lactating [45, 46] periods are critical for efficient progeny generation, by defining the involution period as essential for the timely transition between lactation and dry states. Previous studies have suggested that interference with tsetse fly pregnancy represents a viable target to reduce tsetse populations [2, 10, 43], through the targeting of milk production. This work further describes that interference with post-birth factors also represent targets for novel tsetse control strategies, which modeling suggests can suppress reproduction rates below what is required for population maintenance.

## Materials and methods

### Flies

*Glossina morsitans morsitans* were reared at Yale University and supplemented with those from the Slovak Academy of Sciences. Flies were maintained on bovine blood meals provided through an artificial feeding system at 48h intervals. Tissue samples were collected from pregnant females (16-18d after adult emergence) carrying third instar larvae and at multiple intervals following parturition.

### Evaluation of cellular changes in the milk gland throughout lactation and involution

Images of milk gland cells were acquired with permission from several studies describing the changes in these cells throughout lactation and birth [11–14]. Each image was divided into ten equal quadrants and the area occupied within the milk gland cells by endoplasmic reticulum, Golgi apparti, and autolysosome/lysosomes was measured. Milk gland diameter was measured throughout the course of pregnancy by removing the fat body, uterus and milk gland at various points during the pregnancy cycle. These organs were maintained in PBS (pH 7.4) for less than one hour on ice, after which the diameter of the milk gland was assessed microscopically.

### RNA and protein isolation

RNA and protein isolations were performed using Trizol reagent on whole flies and milk gland/fat body samples following instructions provided by the manufacturer (Invitrogen). RNA was cleaned with an RNeasy Mini Kit (Qiagen). Complementary DNA was synthesized using a Superscript III reverse transcriptase kit from 1μg of the total RNA isolated from each sample.

### quantitative PCR

Transcript levels for *atg8a*, *mgp*, *atg1*, *atg6*, *fk506*-*bp1*, and *diap1* (gene sequences acquired from *Glossina* genome project, vectrobase.org) were determined via qPCR by employing the CFX real-time PCR detection system (Bio-Rad, Hercules, CA) with primers specific to each target gene (S1 Table). All readings were obtained on four biological replicates that were normalized to tsetse *tubulin* expression levels. CFX Manager software version 3.1 (Bio-Rad) was used to quantify transcript expression of each gene and conducted according to methods developed in previous studies [7, 34].

### RNA interference

Short interfering RNA (siRNA) comprised of two duplex sequences (UAAUACGACUCACUAUAGGGACAACGUCAUUCCACCAACA and UAAUACGACUCACUAUAGGGGCCCAGAAAGGGUGUGAAUA) targeting *atg8a* were purchased from IDT in Coralville, IA. Green fluorescent protein (GFP) targeted siRNA (GAUGCCAUUCUUUGGUUUGUCUCCCAU and CUUGACUUCAGCACGUGUCUUGUAGUU) was used as a control.

Previous studies have shown that gene knockdown using siRNA injection yields robust suppression. A spectrophotometer was used to ensure the concentration of each siRNA (*GFP* and *atg8a*) was adjusted to 700–750 ng/μl. siRNA (~1.5μl) was injected into the thorax of tsetse mothers harboring a second instar larva in the uterus. Importantly, previous studies have shown that siRNA injection into the mother does not affect larval transcript levels [7, 10] and as such, any deviations seen in the larva are due to maternal knockdown, not to the unintentional suppression of larval genes. All expression levels were normalized to *tubulin*. Transcript levels were assessed via qPCR as previously described.

### Western blot analysis

Proteins for each time point were extracted in three groups of five flies according to previously described methods [15, 34]. Anti-sera used Include: Rabbit αTubulin (GmmTub, 1:1000) and rabbit α ATG8a (1:1000) were from Bryant and Raikhel [23]. 1/400 of a fly was loaded into each well. Blots were blocked overnight in blocking buffer (PBS, 3% BSA and 0.5% Tween 20, pH 7.4). Exposure time to anti-sera was taken from Attardo et al. [21, 36]. Supersignal West Pico Substrate (Pierce, Wobrun, MA) was used to visualize each blot on an Image Station 2000R (Kodak, New Haven, CT).

### *In situ* hybridization for *atg8a*

Milk gland tubules intertwined with fat body were collected from mothers 0–2 hours after parturition. The combined milk gland/fat body was placed into Carnoy’s fixative for a five-six day fixation period [36]. Antisense/sense digoxigenin-labeled RNA probes were generated using the MAXIscript T7 transcription kit following manufacturer's protocol (Ambion, Austin, TX) using a primer set with a T7 primer (S1 Table) [36]. Antibody solutions were made using α -Digoxigenin-rhodamine Fab fragments (Roche) for FISH probe detection (1:200 dilution) and rabbit α -GmmMGP (1:2500) antibodies [21, 36]. Alexa Fluor 488 goat α -rabbit IgG (Invitrogen) at a dilution of 1:500 was added as a secondary antibody for immunohistochemistry [36]. Slides were mounted in VECTASHIELD Mounting Medium with DAPI (Vector laboratories Inc. Burlingame, CA). Samples were observed using a Zeiss Axioskop2 microscope (Zeiss, Thornwood, NY) equipped with a fluorescent filter and viewed and imaged at 400x magnification. Images were captured using an Infinity1 USB 2.0 camera and software (Lumenera Corporation, Ottawa, Ontario, Canada) and merged in Adobe Photoshop.

### Ecdysone injection

Ecdysone injections were performed according to methods previously described for tsetse flies [32, 47], with modification. Flies used for injection harbored a late first instar or second instar larva, and were in their second reproductive cycle (4–5 days before birth). This is a period when the expression of autophagy genes should be extremely low (This study). Edysone (Sigma-Aldrich) was diluted into 95% ethanol, creating a stock solution of 10 μg/μl. The stock solution was diluted to 1 ng/μl with PBS on the day of injection. Each fly was injected with 0.5 μl. This injection amount is physiologically-relevant based on the increase in ecdysone to ~150 pg/μl noted immediately before birth [22]. RNA was extracted from female flies 24 hours after ecdysone injection, as before. Milk gland width was examined 24 hours after ecdysone treatment. The number of abortions was monitored for three days following ecdysone injection.

### Population modeling

Estimation of the impact of impaired autophagy on population growth, was determined by utilizing a simple model for tsetse population growth modified from Michalkova et al. [43]. The model population for impaired involution-associated autophagy was parametrized with data from the siATG8a treatments. The non-impaired (control) model population was parametrized using data from the siGFP treatment (S2 Table). In each model, mortality was assumed to be normal levels associated tsetse population [48, 49]. We calculated the mean and standard deviation and gonotrophic-cycle length for the control and autophagy-impaired groups over the course of 12 gonotrophic cycles (S2 Table). We modeled fecundity *F*_*jk*_ as a beta random variable with parameters chosen to match the mean and standard deviation of the data. Similarly, gonotrophic-cycle length *t*_*jk*_ was modeled as a log normal random variable with parameters chosen to match the data for each gonotrophic cycle.

Given values of fecundity and gonotrophic cycle length, number of female offspring produced by a single female tsetse over its lifetime is *R*_*j*_ = *p*∑*F*_*jk*_ *S*_*jk*_, where *p* is the probability that a deposited pupa is female, which we took to be 55% [48] and *S*_*jk*_ is the survival, the probability of surviving to gonotrophic cycle *k*. We modeled survival as $S_{jk}\;=\;S_{pupa}s^{T_{jk}}$, where *S*_*pupa*_ is the probability that a deposited pupa survives to emerge as an adult, which we took to be a conservative number at 85% [48, 49]; *s* is the probability of surviving each adult day, which we took to be 98% [48, 49], and *T*_*jk*_ = ∑*t*_*jk*_ is the number of days from emergence until the end of gonotrophic cycle *k*. The population growth rate is then *r*_*j*_ = *R*_*j*_*D*, with the generation time defined to be *D* = *D*_*pupa*_ + *D*_*adult*_; *D*_*pupa*_ is the mean duration of the pupal stage, which we took to be 31.4 days [49]; and *D*_*adult*_ = −[*log*(*s*)]^−1^ is the mean adult lifespan. We calculated the population growth rate *r*_*j*_ for each treatment group and the difference in growth rate between the two treatment groups, *r*_1_ – *r*_2_, for 10,000 samples of our model.

## Supporting information

S1 Table Primers used for qPCR and for *in situ* hybridization analyses. (XLSX)

S2 Table Gonotrophic cycle length and fecundity by cycle number for control and *siatg8a* treatment groups. Cycle length is the duration of the gonotrophic cycle in days. Fecundity is probability that pupa was deposited during the cycle.
(XLSX)

## References

[1] Tobe SS, Langley PA. Reproductive physiology of *Glossina*. Ann Rev Entomol. 1978;23:283–307. doi: 10.1146/annurev.en.23.010178.001435 . 343707 10.1146/annurev.en.23.010178.001435

[2] Benoit JB, Attardo GM, Baumann AA, Michalkova V, Aksoy S. Adenotrophic viviparity in tsetse flies: potential for population control and as an insect model for lactation. Ann Rev Entomol 2015;60:351–357. doi: 10.1146/annurev-ento-010814-020834 . 25341093 10.1146/annurev-ento-010814-020834 PMC4453834

[3] Hagan HR. Embryology of Viviparous Insects. New York Ronald Press; 1951. pmid: N/A

[4] Meier R, Kotrba M, Ferrar P. Ovoviviparity and viviparity in the Diptera. Biol Rev Cambridge Phil Soc. 1999;74(3):199–258. pmid: N/A.

[5] Langley PA, Clutton-Brock TH. Does reproductive investment change with age in tsetse flies, *Glossina morsitans morsitans* (Diptera: Glossinidae)? Funct Ecol 1998;12:866–70. pmid: N/A.

[6] Cmelik SHW, Bursell E, Slack E. Composition of the gut contents of thrid-instar tsetse larvae (*Glossina morsitans* Westwood). Comp Biochem Physiol. 1969;29:447–53. pmid: N/A.

[7] Benoit JB, Hansen IA, Attardo GM, Michalkova V, Mireji PO, Bargul JL, et al Aquaporins are critical for provision of water for lactation and progeny hydration to maintain tsetse fly reproductive success. PLoS Negl Trop Dis. 2014;8:e2517 doi: 10.1371/journal.pntd.0002517 . 24762803 10.1371/journal.pntd.0002517 PMC3998938

[8] International *Glossina* Genome Initiative. Genome sequence of the tsetse fly (*Glossina morsitans*): vector of African trypanosomiasis. Science. 2014;344:380–386. doi: 10.1126/science.1249656 . 24763584 10.1126/science.1249656 PMC4077534

[9] Yang G, Attardo GM, Lohs C, Aksoy S. Molecular characterization of two novel milk proteins in the tsetse fly (*Glossina morsitans morsitans*). Insect Mol Biol. 2010;19:253–62. doi: 10.1111/j.1365-2583.2009.00987.x . 20136662 10.1111/j.1365-2583.2009.00987.x PMC2862765

[10] Benoit JB, Attardo GM, Michalkova V, Bohova J, Zhang Q, Baumann A, et al A novel highly divergent protein family from a viviparous insect identified by RNA-seq analysis: a potential target for tsetse fly-specific abortifacients. PLoS Genetics. 2014;10:e1003874 doi: 10.1371/journal.pgen.1003874 . 24763277 10.1371/journal.pgen.1003874 PMC3998918

[11] Denlinger DL, Ma W-C. Dynamics of the pregnancy cycle in the tsetse *Glossina morsitans*. J Insect Physiol. 1974;20:1015–26. . 4839338 10.1016/0022-1910(74)90143-7

[12] Ma WC, Denlinger DL, Jarlfors U, Smith DS. Structural modulations in the tsetse fly milk gland during a pregnancy cycle. Tissue Cell. 1975;7(2):319–30. . 1145609 10.1016/0040-8166(75)90008-7

[13] Bonnanfant-Jais ML. Morphologie de la gland lactee d-une glossine, *Glossina austeni* Newst, au cours du cycle de gestation. I. Aspects ultrastructuraux en periode de gestation J Micros 1974;19:265–84. pmid: N/A.

[14] Hecker H, Moloo SK. Quantitative morphological changes of the uterine gland cells in relation to physiological events during a pregnancy cycle in *Glossina morsitans morsitans*. J Insect Physiol. 1983;29:651–658. pmid: N/A.

[15] Attardo GM, Benoit JB, Michalkova V, Patrick KR, Krause TB, Aksoy S. The homeodomain protein ladybird late regulates synthesis of milk proteins during pregnancy in the tsetse fly (*Glossina morsitans*). PLoS Negl Trop Dis. 2014;8:e2645 doi: 10.1371/journal.pntd.0002645 . 24763082 10.1371/journal.pntd.0002645 PMC3998940

[16] Zirin J, Perrimon N. *Drosophila* as a model system to study autophagy. Semin Immunopathol. 2010;32:363–372. doi: 10.1007/s00281-010-0223-y 20798940 10.1007/s00281-010-0223-y PMC3562086

[17] Chang YY, Neufeld TP. Autophagy takes flight in *Drosophila*. FEBS Lett. 2010;584:1342–1349. doi: 10.1016/j.febslet.2010.01.006 . 20079355 10.1016/j.febslet.2010.01.006 PMC2843783

[18] Juhasz G, Puskas LG, Komonyi O, Erdi B, Maroy P, Neufeld TP, et al Gene expression profiling identifies FKBP39 as an inhibitor of autophagy in larval *Drosophila* fat body. Cell Death Diff. 2007;14:1181–1190. doi: 10.1038/sj.cdd.4402123 . 17363962 10.1038/sj.cdd.4402123 PMC2084463

[19] Wang SL, Hawkins CJ, Yoo SJ, Muller HA, Hay BA. The *Drosophila* caspase inhibitor DIAP1 is essential for cell survival and is negatively regulated by HID. Cell. 1999;98(4):453–63. PubMed . 10481910 10.1016/s0092-8674(00)81974-1

[20] Yoo SJ, Huh JR, Muro I, Yu H, Wang L, Wang SL, et al Hid, Rpr and Grim negatively regulate DIAP1 levels through distinct mechanisms. Nature Cell Biol. 2002;4:416–424. doi: 10.1038/ncb793 . 12021767 10.1038/ncb793

[21] Attardo GM, Guz N, Strickler-Dinglasan P, Aksoy S. Molecular aspects of viviparous reproductive biology of the tsetse fly (*Glossina morsitans morsitans*): Regulation of yolk and milk gland protein synthesis. J Insect Physiol. 2006;52:1128–36. doi: 10.1016/j.jinsphys.2006.07.007 . 17046784 10.1016/j.jinsphys.2006.07.007 PMC1779500

[22] Robert A, Strambi A, Strambi C. Haemolymph ecdysteriod levels in female tsetse fly *Glossina fuscipes* (Diptera) during the first reproductive cycle: a comparison between and mated females. J Insect Physiol. 1985;32:665–671. pmid: N/A.

[23] Bryant B, Raikhel AS. Programmed autophagy in the fat body of *Aedes aegypti* is required to maintain egg maturation cycles. PLoS One. 2011;6:e25502 doi: 10.1371/journal.pone.0025502 . 22125592 10.1371/journal.pone.0025502 PMC3219638

[24] Raikhel AS, Dhadialla TS. Accumulation of yolk proteins in insect oocytes. Annu Rev Entomol. 1992;37:217–51. doi: 10.1146/annurev.en.37.010192.001245 . 1311540 10.1146/annurev.en.37.010192.001245

[25] Sappington TW, Raikhel AS. Molecular characteristics of insect vitellogenins and vitellogenin receptors. Insect Biochem Mol Biol. 1998;28:277–300. . 9692232 10.1016/s0965-1748(97)00110-0

[26] Riehle MA, Brown MR. Insulin stimulates ecdysteroid production through a conserved signaling cascade in the mosquito *Aedes aegypti*. Insect Biochem Mol Biol. 1999;29:855–60. . 10528406 10.1016/s0965-1748(99)00084-3

[27] Brown MR, Clark KD, Gulia M, Zhao Z, Garczynski SF, Crim JW, et al An insulin-like peptide regulates egg maturation and metabolism in the mosquito *Aedes aegypti*. Proc Natl Acad Sci USA. 2008;105:5716–5721. doi: 10.1073/pnas.0800478105 . 18391205 10.1073/pnas.0800478105 PMC2311378

[28] Brown MR, Graf R, Swiderek KM, Fendley D, Stracker TH, Champagne DE, et al Identification of a steroidogenic neurohormone in female mosquitoes. J Biol Chem. 1998;273:3967–71. . 9461584 10.1074/jbc.273.7.3967

[29] Hansen IA, Attardo GM, Park JH, Peng Q, Raikhel AS. Target of rapamycin-mediated amino acid signaling in mosquito anautogeny. Proc Natl Acad Sci USA. 2004;101:10626–10631. doi: 10.1073/pnas.0403460101 . 15229322 10.1073/pnas.0403460101 PMC489984

[30] Hansen IA, Attardo GM, Roy SG, Raikhel AS. Target of rapamycin-dependent activation of S6 kinase is a central step in the transduction of nutritional signals during egg development in a mosquito. J Biol Chem. 2005;280:20565–72. doi: 10.1074/jbc.M500712200 . 15788394 10.1074/jbc.M500712200

[31] Robert A, Strambi C, Strambi A, Delbecque JP. Ecdysteroids during the development of the tsetse fly. Invert Reprod Develop. 1991;19:71–81. pmid: N/A.

[32] Denlinger DL. Insect hormones as tsetse abortifacients. Nature. 1975;253:347–348. . 1110777 10.1038/253347a0

[33] Robert A, Strambi A, Strambi C, Gonella J. Opposite effects of ecdysone and 20- hydroxyecdysone on *in vitro* uterus motility of a tsetse fly. Life Sci. 1986;39:2617–2622. . 3796208 10.1016/0024-3205(86)90117-7

[34] Benoit JB, Attardo GM, Michalkova V, Takac P, Bohova J, Aksoy S. Sphingomyelinase activity in mother’s milk is essential for juvenile development: a case from lactating tsetse flies. Biol Reprod. 2012; 87:1–10. doi: 10.1095/biolreprod.112.100008 . 22517621 10.1095/biolreprod.112.100008 PMC3406556

[35] Denlinger DL, Ma WC. Maternal nutritive secretions as possible channels for vertical transmission of microorganisms in insects: the tsetse fly example. Ann NY Acad Sci. 1975;266:162–165. . 801109 10.1111/j.1749-6632.1975.tb35097.x

[36] Attardo GM, Lohs C, Heddi A, Alam UH, Yildirim S, Aksoy S. Analysis of milk gland structure and function in *Glossina morsitans*: milk protein production, symbiont populations and fecundity. J Insect Physiol. 2008;54:1236–42. doi: 10.1016/j.jinsphys.2008.06.008 . 18647605 10.1016/j.jinsphys.2008.06.008 PMC2613686

[37] Strickler-Dinglasan PM, Guz N, Attardo G, Aksoy S. Molecular characterization of iron binding proteins from *Glossina morsitans morsitans* (Diptera: Glossinidae). Insect Biochem Molec. 2006;36:921–933. doi: 10.1016/j.ibmb.2006.09.003 . 17098167 10.1016/j.ibmb.2006.09.003 PMC1698469

[38] Guz N, Attardo GM, Wu Y, Aksoy S. Molecular aspects of transferrin expression in the tsetse fly (*Glossina morsitans morsitans*). J Insect Physiol. 2007;53:715–23. doi: 10.1016/j.jinsphys.2007.03.013 . 17498733 10.1016/j.jinsphys.2007.03.013 PMC2065764

[39] Rijnkels M. Multispecies comparison of the casein gene loci and evolution of casein gene family. J Mammary Gland Biol Neoplasia. 2002;7:327–45. . 12751895 10.1023/a:1022808918013

[40] Ginger MR, Grigor MR. Comparative aspects of milk caseins. Comp Biochem and Physiol B-Biochem & Mol Biol. 1999;124:133–145. pmid: 12751895. 10584297 10.1016/s0305-0491(99)00110-8

[41] Watson CJ, Kreuzaler PA. Remodeling mechanisms of the mammary gland during involution. Int J Dev Biol. 2011;55:757–762. doi: 10.1387/ijdb.113414cw . 22161832 10.1387/ijdb.113414cw

[42] Gajewska M, Zielniok K, Motyl T. Autophagy in development and remodelling of mammary gland In: Bailly Y, editor. Autophagy—A Double-Edged Sword—Cell Survival or Death?: InTech 2013.

[43] Michalkova V, Benoit JB, Attardo GM, Medlock J, Aksoy S. Amelioration of reproduction-associated oxidative stress in a viviparous insect is critical to prevent reproductive senescence. PLoS One. 2014;24:e87554 doi: 10.1371/journal.pone.0087554 pmid: 24763119 10.1371/journal.pone.0087554 PMC3998933 24763119

[44] Hu CY, Rio RVM, Medlock J, Haines LR, Nayduch D, Savage AF, et al Infections with immunogenic trypanosomes reduce tsetse reproductive fitness: potential impact of different parasite strains on vector population structure. PLoS Negl Trop Dis. 2008;12:e192 doi: 10.1371/journal.pntd.0000192 pmid: 18335067. 10.1371/journal.pntd.0000192 PMC2265429 18335067

[45] Attardo GM, Benoit JB, Michalkova V, Yang G, Roller L, Bohova J, et al Analysis of lipolysis underlying lactation in the tsetse fly, *Glossina morsitans*. Insect Biochem Mol Biol. 2012;42:360–70. . 22509523 10.1016/j.ibmb.2012.01.007 PMC3561780

[46] Baumann A, Benoit JB, Michalkova V, Mireji PO, Attardo GM, Moulton JK, et al Juvenile hormone and insulin suppress lipolysis between periods of lactation during tsetse fly pregnancy. Mol Cell Endocrinol. 2013;372:30–41. doi: 10.1016/j.mce.2013.02.019 . 23499946 10.1016/j.mce.2013.02.019 PMC4222070

[47] Zdarek J, Denlinger DL. Neural regulation of pupariation in tsetse larvae. J Exp Biol. 1992;173:11–24. . 1487712 10.1242/jeb.173.1.11

[48] Madubunyi LC. Survival and productivity of the tsetse, *Glossina morsitans morsitans* Westwood (Diptera:Glossinidae) maintained udner different feeding regimens through four successive reproductive cycles in Zambia. Insect Sci Appl. 1989;10:75–80. pmid:N/A.

[49] Jarry M, Khaladi M, Gouteaux JP. A matrix model for studying tsetse fly population. Entomol Exp Appl. 1996;78:51–60. pmid:N/A.

